# Myocardial injury in a 41-year-old male treated with methylphenidate: a case report

**DOI:** 10.1186/1756-0500-7-480

**Published:** 2014-07-29

**Authors:** Lisa Drange Hole, Jan Schjøtt

**Affiliations:** 1 Section of Clinical Pharmacology, Laboratory of Clinical Biochemistry, Haukeland University Hospital, 5021 Bergen, Norway; 2 Institute of Clinical Science, Faculty of Medicine and Dentistry, University of Bergen, Bergen, Norway; 3 Regional Medicines Information and Pharmacovigilance Centre (RELIS Vest), Haukeland University Hospital, 5021 Bergen, Norway

**Keywords:** Supraventricular tachycardia, Troponin, Methylphenidate, Venlafaxine, Acute coronary syndrome, Coronary artery spasm, Cardiac magnetic resonance imaging

## Abstract

**Background:**

Elevated cardiac troponin levels are consistent with the diagnosis of an acute coronary syndrome, but may also represent adverse drug reactions. Psychostimulating drugs raise both blood pressure and heart rate, and case reports of sudden death, stroke, and myocardial infarction have led to regulatory and public concern about the cardiovascular safety of these drugs.

**Case presentation:**

We present a case where a 41-year-old Norwegian male with radiating chest pain, elevated troponins, and supraventricular tachycardia was hospitalized. Tentative diagnosis was acute coronary syndrome. Percutaneous coronary angiography, but not cardiac magnetic resonance imaging, was performed and medical antiplatelet treatment started. Because of an attention deficit/hyperactivity disorder the patient had recently increased his dose of methylphenidate, but still within the therapeutic dose range. Apart from venlafaxine, also in a therapeutic dose, the patient took no other drugs. An acute coronary syndrome was excluded during hospitalization, and a drug effect was suspected.

**Conclusions:**

When interpreting troponin results it is important to take into account the context of the patient’s clinical presentation, including the possibility of adverse drug reactions. The adverse drug reaction could include a combination of vasospasm and/or increased oxygen demand due to tachycardia. This case should be borne in mind before a diagnosis of myocardial infarction is given, or a decision to perform invasive coronary angiography is made in patients that use methylphenidate or related substances. Cardiac magnetic resonance imaging could be of diagnostic value in such cases.

## Background

Elevated cardiac troponin levels are consistent with the diagnosis of an acute coronary syndrome, but may also represent adverse drug reactions. Psychostimulating drugs raise both blood pressure and heart rate, and case reports of sudden death, stroke, and myocardial infarction (MI) have led to regulatory and public concern about the cardiovascular safety of these drugs [1]. Methylphenidate (Ritalin®) is used for the treatment of attention-deficit hyperactivity disorder (ADHD) and misused recreationally and as a cognitive enhancer. Methylphenidate inhibits the dopamine and norepinephrine transporters thereby elevating dopamine and norepinephrine levels in the brain [2]. In the Summary of Product Characteristics (SPC) for Ritalin®*,* tachycardia is reported as a common adverse reaction (≥1/100 to < 1/10), chest pain is reported as less common (≥1/1000 to < 1/100), angina pectoris is reported as a rare adverse reaction (≥1/10 000 to < 1/1000), and supraventricular tachycardia (SVT) and MI are reported as *<* 1/10 000 or unknown. Venlafaxine (Efexor Depot®) is a selective monoamine reuptake inhibitor. In the SPC for Efexor Depot®, tachycardia is reported as a less common adverse reaction (≥1/1000 to < 1/100), ventricular tachycardia and torsades de pointes are reported as rare (≥1/10 000 to < 1/1000) [3]. The Regional Medicines Information and Pharmacovigilance Centre (RELIS) in Norway have the responsibility for regional pharmacovigilance based on a spontaneous adverse drug reaction reporting system. The present report was submitted to RELIS in September 2013.

The report represented a suspected, but not confirmed, acute coronary syndrome in a 41-year-old male treated with methylphenidate. The presentation of the case follows the guidelines for submitting adverse drug reaction for publication [4].

## Case presentation

Case report: A 41-year-old Norwegian male with ADHD was admitted to hospital because of tachycardia, chest pain and radiation of pain to his right arm and neck. A week prior to hospitalization his general practitioner increased the patient’s Ritalin® dose from 60 to 80 mg daily plus three 10 mg tablets, if needed, per day. The patient also used 75 mg of Efexor Depot® × 1 daily because of anxiety. Previous drug abuse was reported, but the patient was now only taking prescribed medication. The patient had previously experienced heart palpitations, but never persistent tachycardia. He had never been hospitalized before. The tachycardia and chest pain commenced while the patient was seated, not in relation to increased physical activity, and lasted approximately two hours, with concomitant nausea. At admission, blood pressure was 113/79 mmHg and heart rate was 200 bpm. Electrocardiogram (ECG) showed an SVT with a frequency of 200 beats per minute (bpm). There was no ST-segment elevation. Cardiac troponin I (cTnI) was 82 (<30 ng/L), 6 hours later 616 and 12 hours later 529. Creatine kinase (CK) was 10 (<5 ug/L). Carotic massage had no effect on his tachycardia. However, the patient converted to sinus rhythm spontaneously 15 minutes after arriving at the hospital. At conversion to sinus rhythm, his ECG was still without ST-segment elevation. In accordance with the European Society of Cardiology guidelines for the management of acute coronary syndromes in patients presenting without persistent ST-segment elevation [5], the patient was given 300 mg acetylsalicylic acid (Acetylsalisylsyre®) and 180 mg ticagrelor (Brilique®) orally as a starting dose, and the following two days before dismissal from the hospital 75 mg acetylsalicylic acid and 90 mg ticagrelor x 2 per day and 10 mg atorvastatin (Lipitor®). The patient was observed, and reported no further pain or distress. Because of elevated cTnI and CK levels and because of his cardiovascular risk factors of heritage, smoking, gender and clinical presentation, further examinations were carried out: Cardiac ultrasound showed minimal hypokinesia in the proximal lower wall of the left ventricle, but adequate systolic function with an ejection fraction (EF) > 60% (normal). Percutaneous coronary angiography was performed showing no pathology. Acetylcholine provocation test was not performed during the angiography. No further cardiac investigations, including cardiac magnetic resonance imaging (CMR), were performed. SVT and elevated troponin levels were interpreted as related to ingestion of methylphenidate and venlafaxine. His methylphenidate dose was reduced to 30 mg times 2 daily, with no additional 10 mg tablets. The patient was dismissed from hospital.

## Discussion

Methylphenidate is used in treatment of ADHD from the age of six. In Norway, methylphenidate is not approved as treatment for adults, because safety and effect of the drug is not documented according to the SPC. However, most guidelines recommend methylphenidate as first-line medication for ADHD in adults [6]. When consulting Embase and PubMed, no interactions are reported between venlafaxine and methylphenidate. In the present case an acute coronary syndrome was excluded, and we believe his troponin rise and symptoms could be an adverse drug reaction. Maximum dose methylphenidate is 60 mg/day in children and 80–100 mg/day in adults. However, an exact maximum dose has not been determined. Recommended starting dose of venlafaxine depot capsules is 75 mg as a single dose daily. Patients that do not respond to the starting dose might benefit from an increased dose, up to a maximum dose of 225 mg/day. Our patient states retrospectively that he took 40 mg methylphenidate in the morning and 40 mg at night. He did not take any 10 mg tablets of methylphenidate the week prior to hospitalization. Both methylphenidate and venlafaxine have been associated with cardiovascular effects, including tachycardia. Furthermore, one cannot rule out a pharmacodynamic interaction between the two drugs for the SVT and troponin elevation. One study investigated whether use of methylphenidate in adults is associated with elevated rates of serious cardiovascular events compared with rates in nonusers. Although initiation of methylphenidate was associated with a 1.8-fold increase in risk of sudden death or ventricular arrhythmia, the lack of a dose–response relationship suggests that this association may not be a causal one [1].

One case report describes a man, aged 27, whom suffered an acute anterolateral MI related to use of prescribed methylphenidate (5 mg twice daily for adult attention deficit disorder) [7]. During the night preceding his hospitalization, the patient had taken three extra 5 mg doses (totaling 25 mg over a 24 hour period) of methylphenidate. Blood pressure, pulse, respiratory frequency and temperature were normal, however ECG showed > 1 mm ST-elevation in leads I, AVL, V5 and V6. cTnI was 9.32 ng/mL. Cardiac catheterization was normal, but there was severe inferolateral hypokinesia, consistent with MI. The cardiology service believed that the acute MI was due to vasospasm caused by methylphenidate. The authors of this case report suggest that treatment of this type of MI should be somewhat different from the usual treatment given to patients with coronary artery disease, because these events are more likely to occur in a younger population without significant coronary artery disease, and the mechanism is likely to be due to coronary artery vasospasm [7]. Based on this observation and on our case report, one might argue that sympathomimetic-induced MI should be treated with aspirin, nitrates and benzodiazepines initially, until the underlying cause of myocardial ischemia has been resolved [8].

Coronary artery spasm is an important cause of myocardial ischemia, and can lead to acute MI, ventricular arrhythmias and sudden death [9]. The pathological mechanisms leading to coronary artery spasm are not fully understood. The degree of vasoconstriction can vary from complete occlusion to minimal contraction of an artery segment. Ischemia occurs when the oxygen demand in the myocardium becomes larger than the oxygen supply. Coronary artery spasm is known to be caused by hyperventilation, cocain, tobacco, histamin, serotonin and acetylcholine [10].

Regarding methylphenidate, it seems plausible that the combination of adrenergic and cholinergic actions of this drug may disrupt the coronary autonomic balance in favor of coronary vasoconstriction [11]. A dynamic stenosis associated with such a coronary vasospasm can limit the supply of oxygen and nutrients and, in the worst case, precipitate MI.

The diagnosis of coronary vasospasm is based upon the angiographic finding of epicardial coronary narrowing that is promptly reversed by vasodilators. If normal coronaries are found at angiography, the diagnosis usually becomes one of exclusion based on clinical and electrocardiographic criteria [11]. A coronary spasm provocation test can be performed. The test involves intracoronary injection of acetylcholine in patients who show no culprit lesion on coronary angiography and are suspected to have coronary spasm as the cause of chest pain [12]. In our case the coronary angiography was negative. An acetyl choline provocation test was not performed, which is a limitation to our case report. Acetyl choline provocation tests are not routinely performed in Norwegian hospitals. Cardiac ultrasound showed minimal hypokinesia in the proximal lower wall of the left ventricle, but adequate systolic function with a normal EF. His ECG showed no signs of ongoing ischemia, but an SVT with a frequency of 200 bpm. cTnI elevation might be caused by the SVT or an episode of coronary artery spasm that occurred, and remitted spontaneously, before angiography was performed. This might explain the minimal hypokinesia caught on the cardiac ultrasound, however, not depicted on the ECG.

A consensus statement published to give a universal definition of the term MI, classifies MI into various subtypes [13]: Type 1 is a spontaneous MI related to ischemia from a primary coronary event (e.g., plaque rupture, thrombotic occlusion). Type 2 is secondary to ischemia from a supply-and-demand mismatch. Type 3 is an MI resulting in sudden cardiac death. Type 4a is an MI associated with percutaneous coronary intervention, and 4b is associated with in-stent thrombosis. Type 5 is an MI associated with coronary artery bypass surgery. According to this consensus, our patient experienced a type 2 MI, where SVT and/or a dynamic stenosis associated with a coronary vasospasm limited the supply of oxygen and nutrients, or both. Some patients with SVT have increased troponin levels without signs of coronary artery disease [14]. The exact mechanism of myocardial injury and elevated troponin release in SVT is unclear. It may be due to an increase in oxygen consumption by the myocardium during tachycardia. Also, a reduction of the oxygen supply to the myocardium due to the shortened diastole with subsequent subendocardial ischemia during tachycardia is possible [15]. Although cTnI elevation in patients presenting to the hospital with SVT is well recognized, the prevalence, predictors, and prognostic significance of cTnI elevation associated with SVT presentation are not known. Mild elevation of cTnI is common in patients presenting to the hospital with SVT, and is associated with increased risk of future cardiovascular events [16].

In most patients, either conservative management or noninvasive stratification seems to be sufficient; an invasive strategy could then be reserved only for high-risk patients [15]. The presence of tachycardia sufficient to warrant hospital admission can raise troponin, and this should be viewed in context when a decision on angiography is to be taken [17]. In cases where methylphenidate is suspected to have caused coronary artery spasm, the medical treatment should consist of coronary vasodilators, such as nitrates and calcium channel-blockers [10,11]. Furthermore, CMR thanks to high spatial resolution and tissue characterization, can be crucial in identifying myocardial damage and its endocardial (ischemic damage) or midwall/epicardial localization (non ischemic damage). Particularly, T2 weighted images permit identification of myocardial damage. Delayed contrast enhancement permits identification of the extent of scar tissue. In the absence of a definite and clear diagnosis of disease, we think that the identification of damage location is necessary to confirm myocardial damage due to coronary disease (acute MI with normal coronary arteries) or to identify damage due to methylphenidate. The lack of a CMR scan is a limitation in our case report. Our department received the adverse effect report from the physician three weeks after the patient was dismissed from the hospital. Routinely, serum is only kept for 8 days after dismissal, therefore, serum measurements of methylphenidate or venlafaxine was not available.

## Conclusion

To our knowledge this is the first case report in which the use of methylphenidate, in combination with venlafaxine, both in therapeutic doses, is associated with high levels of cardiac troponin, suspicious of acute coronary syndrome. We speculate that methylphenidate could give this effect based on its arrthymogenic potential in higher doses, its potential to cause coronary artery vasospasms, or both. Our case should be kept in mind before a diagnosis of infarction is given or decision to perform coronary angiography is made, in patients who use methylphenidate or related substances. CMR could be of diagnostic value in such cases.

## Consent

Written informed consent was obtained from the patient for publication of this Case Report and any accompanying images. A copy of the written consent is available for review by the Editor-in-Chief of this journal.

## Competing interest

The authors declare that they have no competing interests.

## Authors' contributions

LDH conceived of the case report, participated in the design and coordination of the case report, collected the data and the informed consensus, and wrote the manuscript. JS conceived of the case report, participated in the design of the case report, and helped to draft and co-write the manuscript and supervised all aspects of the case report. All authors read and approved the final manuscript.
